# Mutation accumulation and developmental lineages in normal and Down syndrome human fetal haematopoiesis

**DOI:** 10.1038/s41598-020-69822-1

**Published:** 2020-07-31

**Authors:** Karlijn A. L. Hasaart, Freek Manders, Marie-Louise van der Hoorn, Mark Verheul, Tomasz Poplonski, Ewart Kuijk, Susana M. Chuva de Sousa Lopes, Ruben van Boxtel

**Affiliations:** 1 Princess Máxima Center for Pediatric Oncology and Oncode Institute, Heidelberglaan 25, 3584CS Utrecht, The Netherlands; 2 Leiden University Medical Center, 2333 ZC Leiden, The Netherlands; 3 Princess Máxima Center for Pediatric Oncology, Heidelberglaan 25, 3584CS Utrecht, The Netherlands; 4 Center for Molecular Medicine, University Medical Center Utrecht and Oncode Institute, Universiteitsweg 100, 3584 CG Utrecht, The Netherlands; 5 Department of Anatomy and Embryology, Leiden University Medical Center, 2333 ZC Leiden, The Netherlands

**Keywords:** Cancer, Developmental biology, Genetics

## Abstract

Children show a higher incidence of leukemia compared to young adolescents, yet their cells have less age-related (oncogenic) somatic mutations. Newborns with Down syndrome have an even higher risk of developing leukemia, which is thought to be driven by mutations that accumulate during fetal development. To characterize mutation accumulation in individual stem and progenitor cells of Down syndrome and karyotypically normal fetuses, we clonally expanded single cells and performed whole-genome sequencing. We found a higher mutation rate in haematopoietic stem and progenitor cells during fetal development compared to the post-infant rate. In fetal trisomy 21 cells the number of somatic mutations is even further increased, which was already apparent during the first cell divisions of embryogenesis before gastrulation. The number and types of mutations in fetal trisomy 21 haematopoietic stem and progenitor cells were similar to those in Down syndrome-associated myeloid preleukemia and could be attributed to mutational processes that were active during normal fetal haematopoiesis. Finally, we found that the contribution of early embryonic cells to human fetal tissues can vary considerably between individuals. The increased mutation rates found in this study, may contribute to the increased risk of leukemia early during life and the higher incidence of leukemia in Down syndrome.

## Introduction

The initiation and progression of cancer is thought to result from somatic clonal evolution in human tissues^1^. DNA mutations promote heritable phenotypic diversity in cell populations, which provides the substrate for context-dependent selection forces. Oncogenic mutations allow cells to become independent of external growth factors, or insensitive to intrinsic inhibitory signals, which in the correct context can promote uncontrolled clonal expansion and eventually cancer. For adult cancers, the acquisition of oncogenic mutations is thought to be rate limiting for tumor initiation, providing an explanation why aging is the biggest risk factor for developing cancer^2,3^. Indeed, somatic mutations accumulate gradually throughout human life^4,5^. However, children can also develop cancer. In fact, for some cancers, such as leukemia, the incidence is higher in children compared to adolescents, even though their young cells have less age-related (oncogenic) mutations^6^. The mutations driving pediatric leukemia are thought to be acquired during fetal development^7^; however, the rate and patterns of mutation accumulation in haematopoietic stem and progenitor cells (HSPCs) during fetal development are currently not known.

Newborns with Down syndrome (DS) provide an opportunity to better understand the molecular mechanisms underlying pediatric leukemogenesis, because DS children show a substantially elevated risk of developing leukemia during their first years of life^8^. Children with DS have a 500 fold higher risk of developing acute megakaryoblastic leukemia (DS-AMKL) compared to the general population^8,9^. DS-AMKL is often preceded by DS-associated myeloid preleukemia, which is observed in 5–10% of all DS newborns and usually spontaneously disappears within the first 3–4 months after birth^9^. However, even when spontaneous regression is achieved, approximately 20–30% of all DS-associated myeloid preleukemia patients will develop DS-AMKL^10^. This suggests that an extra copy of chromosome 21 can act as a genetic driver of cancer, but that additional oncogenic driver mutations are required^11,12^. In line with this, DS-associated myeloid preleukemia is characterized by somatic mutations in *GATA1*, which cause a N-terminally truncated protein^13^. These *GATA1* mutations are acquired during fetal development and are sufficient for the development of DS-associated myeloid preleukemia^13,14^. Remarkably, it has been reported that in some DS-associated myeloid preleukemia patients several independent clones exist, which are characterized by distinct *GATA1* mutations^12^. This observation suggests that the HSPCs in the fetal liver of DS fetuses might be subjected to high levels of mutagenesis. Previously, it has been shown that aneuploidy in yeast results in genomic instability^15^. However, it is not known if an aneuploidy of chromosome 21 causes an increase in somatic mutation load in cells of human trisomy 21 (T21) fetuses. To compare the somatic mutation rates and patterns during normal and T21 fetal development, we studied mutation accumulation in single HSPCs and intestinal stem cells (ISCs) of fetuses with a normal karyotype and of fetuses with T21. We found an increased somatic mutation rate in fetal HSPCs and even higher somatic mutation numbers in cells of T21 fetuses. Moreover, we found that somatic mutations in DS-associated preleukemia can be explained by mutational processes, which are normally active in normal and T21 fetal haematopoiesis. Second, we showed that the contribution of developmental lineage branches to fetal tissues can be symmetric as well as asymmetric. This observation indicates that the contribution of developmental lineage branches to tissues can vary between fetuses, independent of T21.

## Results

### Mutation accumulation during human fetal haematopoiesis

**Figure 1:**
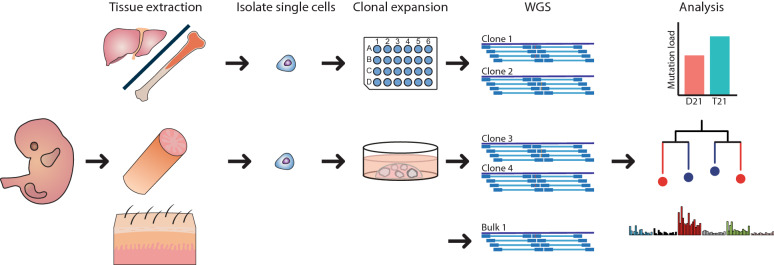
Characterizing somatic mutations in single fetal haematopoietic stem and progenitor cells (HSPCs) and fetal intestinal stem cells (ISCs). (**a**) Experimental strategy for characterizing somatic mutations in single cells of disomy 21 (D21) and trisomy (T21) fetuses. HSPCs and ISC were clonally expanded to obtain sufficient DNA for whole-genome sequencing (WGS). DNA from bulk skin or intestine was used as a reference to control for germline variants. After characterizing the somatic mutations in single cells, the somatic mutation load between D21 and T21 fetal cells was compared. In addition, signature analysis and phylogenetic lineage tree analyses were performed.

**Figure 2:**
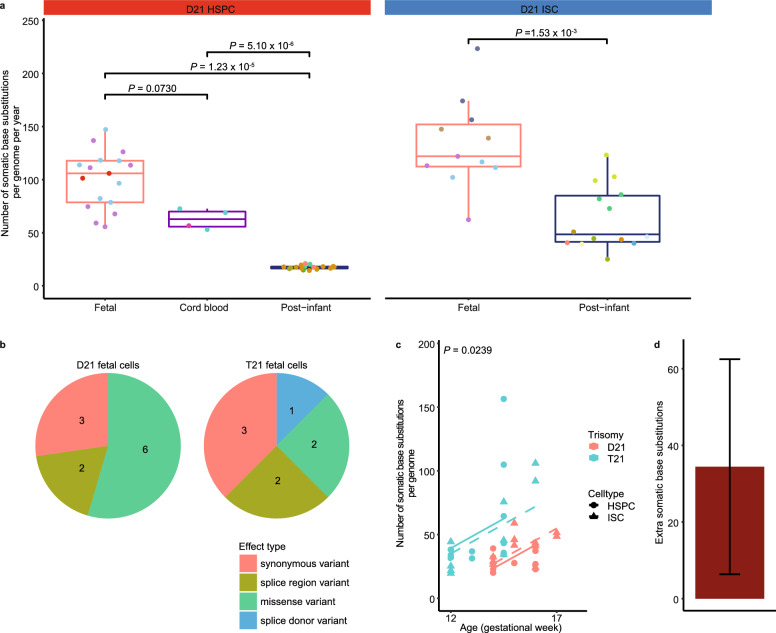
Accumulation of somatic base pair substitutions in haematopoietic stem and progenitor cells (HSPCs) and intestinal stem cells (ISCs) during human fetal development and after birth**.** (**a**) Comparison of the number of autosomal somatic base substitutions per genome per year between D21 HSPCs (D21 fetal: 17 clones; 3 donors, Cord blood: 4 clones; 2 donors, Post-infant: 18 clones; 6 donors) and D21 ISCs (D21 fetal: 11 clones; 4 donors, Post-infant: 14 clones; 9 donors) of fetuses, cord blood and post infant (linear mixed-effects model). Points with the same color indicate single cells from the same subject. (**b**) Pie charts showing the number of somatic mutations for different types of exonic mutations in D21 and T21 fetal stem and progenitor cells (D21 fetal: 28 clones; 5 donors, T21 fetal: 23 clones; 4 donors). **c** The number of somatic base substitutions per genome plotted against the donor age (D21 fetal: 28 clones; 5 donors, T21 fetal: 23 clones; 4 donors). Dashed line: ISC, full line: HSPC. P-value shows the difference between T21 and D21 fetal stem and progenitor cells. (linear mixed-effects model, two-tailed t-test). **d** Extra somatic base substitutions per genome in T21 fetal stem and progenitor cells. Error bars represent 95% confidence intervals.

Cataloguing somatic mutations in physiologically normal cells is technically challenging due to the polyclonal nature of healthy tissues and the high error rate of single cell sequencing techniques^16^. Previously, we have developed a method to characterize somatic mutations in single cells using clonal cultures of primary human stem cells of various tissues^17^, including adult HSPCs^4^. Here, we applied a similar approach to catalogue somatic mutations in fetal HSPCs as well as donor-matched ISCs (Fig. 1). We included 9 independent human fetuses gestational age (GA) week 12–17) (Supplementary Table S1 online). Four of these fetuses had a constitutive T21 and five of these fetuses were karyotypically normal (D21) (Supplementary Table S1 online). We isolated HSPCs (CD34+, lineage−) from liver and bone marrow (Supplementary Fig. S1 online) and clonally expanded these cells for 3–4 weeks in culture to obtain sufficient DNA for whole-genome sequencing (WGS)^18^. Moreover, we clonally expanded ISCs of the same fetus into organoid cultures for 6–7 weeks and performed WGS. From each fetus, we sequenced DNA from bulk skin or intestine to control for germline variants (see Methods). This approach allowed us to obtain all the mutations that were present in the originally expanded fetal stem and progenitor cells and which were acquired in vivo^17,18^. Mutations that accumulated during the in vitro expansion could be excluded based on their low variant allele frequency (VAF) (Supplementary Fig. S2 online), as not all the cells in the clonal culture share these mutations in contrast to the in vivo acquired mutations. In total, we observed 740 base substitutions and 42 indels in 17 clonal D21 HSPCs and 11 clonal D21 ISC cultures, which were obtained from 5 independent fetuses (Fig. 2a, Supplementary Table S2 online). In addition, we found 873 base substitutions and 41 indels in 14 clonal T21 HSPCs and 9 clonal T21 ISC cultures obtained from 4 independent fetuses (Supplementary Table S2 online). We did not observe any larger structural variants or chromosomal aberrations (see Methods). Almost all somatic mutations were located in introns. In total we found 11 somatic mutations located in exons in D21 fetal stem and progenitor cells and 8 in T21 fetal stem and progenitor cells, none of which we considered to be drivers (Supplementary table S3 online) (see Methods). Moreover, we did not observe a mutation in *GATA1* in any of the fetal stem and progenitor cells, suggesting that there is no myeloid preleukemia clone present. There was no significant difference in the types of somatic exonic mutations between D21 and T21 fetal stem and progenitor cells (*P* = 0.578, chi-squared test) (Fig. 2b). In addition, we compared our data to genome-wide mutation catalogues observed in D21 post-infant HSPCs and D21 post-infant ISCs obtained from our previous studies^4,5^. We calculated the somatic mutation rate of HSPCs and ISCs during fetal development and after birth by dividing the number of somatic mutations by the age (in years) of the fetus or donor since conception. We observed an annual somatic mutation rate in D21 fetal HSPCs of approximately 100 base substitutions per year (95% confidence interval: 88–113), which is 5.8 times higher compared to the rate observed in D21 post-infant HSPCs (*P* = 1.231 × 10^–5^, linear mixed-effects model) (Fig. 2a). We also observed a higher mutation rate in D21 fetal ISCs compared to D21 post-infant ISCs (*P* = 0.00153, linear mixed-effects model) (Fig. 2a), which is line with a previous study that catalogued somatic mutations in D21 fetal ISCs^19^.

### Increased mutation load during fetal development in Down syndrome

T21 stem and progenitor cells of T21 fetuses accumulated about 34 (95% confidence interval: 6–62) extra somatic base pair substitutions mutations per cell compared to D21 stem and progenitor cells during fetal development (*P* = 0.0239, linear mixed-effects model) (Fig. 2c,d; Supplementary Fig. S3, S4 online). Of note, this increase was not restricted to the haematopoietic system. We validated that the difference in mutation load between T21 and D21 fetal stem and progenitor cells was not dependent on one or even two single data points (Supplementary Fig. S4 online), underlining the robustness of our finding. Interestingly, T21 fetal stem and progenitor cells also showed an increased variance in somatic mutation load compared to D21 fetal cells (*P* = 2.1 × 10^–8^, likelihood-ratio test, LR: 31) with some cells showing 2–3 times higher mutation load than age-matched D21 fetal cells (Fig. 2c). Of these, one HSPC of a GA week 14.5 T21 fetus showed a significantly higher number of somatic mutations than expected compared to other T21 fetal stem and progenitor cells (linear mixed-effects model, two-sided outlier test, FDR = 0.049) (Fig. 2c). Finally, we observed 9 double base pair substitution (DBS) in T21 fetal stem and progenitor cells versus only 1 DBS in D21 fetal stem and progenitor cells (*P* = 0.0017, Wilcoxon test) (Supplementary Fig. S5 online). We did not observe a difference in the number of indels between T21 and D21 fetal stem and progenitor cells (*P* = 0.815, linear mixed-effects model) (Supplementary Fig. S6 online). Taken together, our results show that the presence of a constitutive T21 in T21 fetuses results in an increased number of base substitutions as well as an increased variance in mutation load between different stem and progenitor cells.

### Mutation accumulation during early embryogenesis

**Figure 3:**
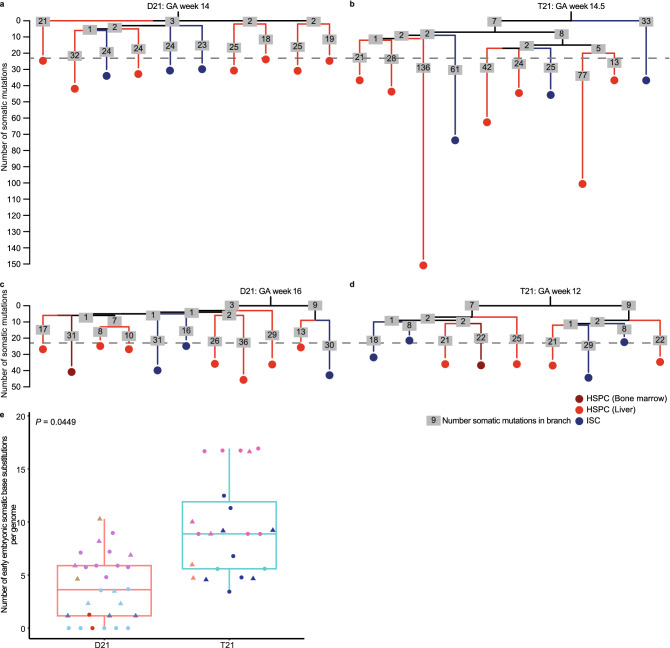
Phylogenetic lineage trees of disomy 21 (D21) and trisomy 21 (T21) fetuses. (**a**) Lineage trees of a gestational age (GA) week 14 D21 fetus, **b** gestational age week 14,5 T21 fetus,** c** gestational age week 16 D21 fetus and **d** gestational age week 12 T21 fetus. Each tip represents a single clonally expanded cell. The length of the branches indicates the number of somatic mutations in that branch of the tree. The number of somatic mutations in each branch are shown in grey boxes. **e** Comparison of the number of somatic base substitutions per genome between D21 and T21 fetal stem and progenitor cells, that occurred early in the development of the fetus (D21 fetal: 28 clones; 5 donors, T21 fetal: 23 clones; 4 donors). Circle: Haematopoietic stem and progenitor cells, Triangle: intestinal stem cells. Points with the same color indicate single cells from the same subject. (linear mixed-effects model, two-tailed t-test).

To determine when during fetal development the difference in mutation load between T21 and D21 fetal stem and progenitor cells occurred, we used a phylogenetic analysis approach to time the occurrence of somatic mutations during development (see Methods). Somatic mutations that are shared between two fetal stem and progenitor cells reflect a historical common ancestor. The more mutations two cells share, the later during development these two cells separated from a common ancestral cell^20,21^. By assessing all the mutations that are shared between the different cells of the same fetus, we constructed developmental lineage trees for 2 D21 and 2 T21 fetuses (Fig. 3a–d). Mutations near the trunk of the developmental lineage tree are shared between endoderm-derived ISCs and mesoderm-derived HSPCs, indicating that these mutations were acquired before gastrulation. In line with this, these mutations also showed sub-clonal presence in the matching skin bulk sample, which is derived from ectoderm. We used this analysis to compare the mutation rates during early embryonic development between T21 and D21 fetuses (see Methods). We found about 6 (95% confidence interval: 0.2–11.7) extra somatic mutations per cell acquired during the first cell divisions in T21 fetal stem and progenitor cells compared to D21 fetal stem and progenitor cells (*P* = 0.0449, linear mixed-effects model) (Fig. 3e). This observation indicates that the mutation load is already increased in T21 very early after conception, before gastrulation.

### Contribution of developmental lineage branches to D21 and T21 fetal tissues

**Figure 4:**
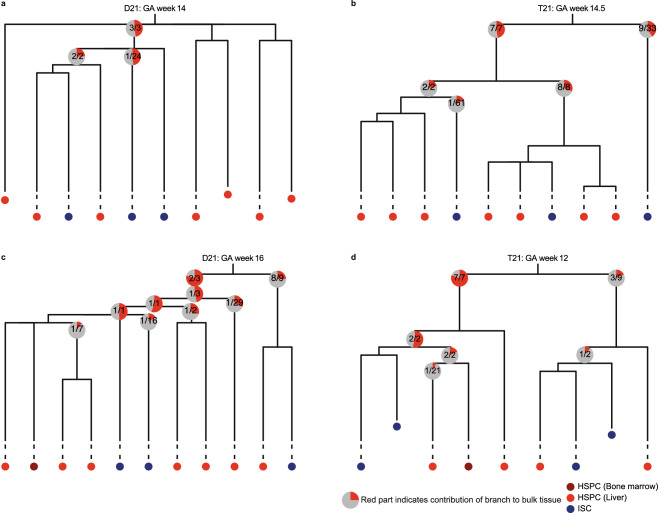
Relative contribution of the developmental lineage branches to fetal skin tissue. (**a**) Lineage trees of a gestational age (GA) week 14 D21 fetus, (**b**) gestational age week 14,5 T21 fetus, (**c**) gestational age week 16 D21 fetus and (**d**) gestational age week 12 T21 fetus. Each tip represents a single clonally expanded cell. The pie charts show the median contribution of the contributing mutations in a branch to the bulk skin tissue. The grey part of the pie chart indicates the total skin tissue, while the red part shows the contribution of a single branch to the skin tissue. The text in the pie charts shows how many of the mutations in that branch contributed to the skin tissue. Mutations not contributing to the skin tissue at all, are not used to calculate the median. Multiple pie charts in a single branch indicate that the mutations in that branch occurred during different cell divisions.

We used the developmental lineage trees to study the contribution of early embryonic branches to bulk skin in each fetus. For this analysis, we compared for each fetus the median VAF in bulk skin of the somatic mutations accumulated before gastrulation between the first 2 developmental lineages branches (Fig. 4). Because all somatic mutations are accumulated during the same period of fetal development, we were able to compare fetuses of different GA. Previous studies using similar mutational analyses in adult HSPCs obtained from human donors revealed an asymmetric contribution of developmental branches to the adult haematopoietic system^4,21,22^. Also in adult mice, this asymmetric contribution of developmental branches was observed^20^. These observations indicate that early embryonic cells do not contribute equally to adult tissues. In line with this, we found that the phylogenetic lineage trees of a GA week 16 D21 fetus and a GA week 12 T21 fetus showed an asymmetric contribution of the first 2 detectable developmental lineage branches (*P* = 2.259 × 10^–13^, 1.525 × 10^–13^, chi-squared test) (Fig. 4c,d). However, we did not observe this asymmetric contribution in a GA week 14.5 T21 and a GA week 14 D21 fetus (Fig. 4a,b). This observation indicates that the contribution of early embryonic cells to fetal tissues can vary between fetuses, independent of T21.

### Activity of mutational processes in post-infant, D21 and T21 fetal haematopoiesis

**Figure 5:**
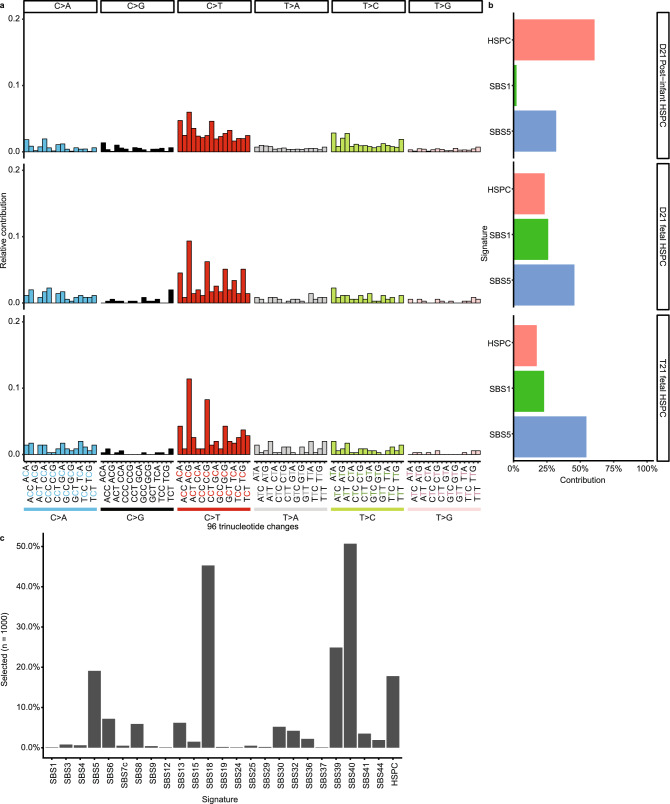
Somatic mutation patterns of disomy 21 (D21) and Trisomy 21 (T21) haematopoietic stem and progenitor cells (HSPCs). (**a**) Spectra of somatic point substitutions. The substitutions are pooled per category. (D21 Post-infant HSPC: n = 10,924; 18 clones; 5 donors, D21 fetal HSPC: n = 353; 17 clones; 3 donors, T21 fetal HSPC: n = 351; 13 clones; 3 donors). (**b**) The relative contribution of each mutational signature to the spectra of point substitutions. (**c**) Bar plot depicting how often each signature was selected during a bootstrapped (1000 iterations) signature selection process for the T21 fetal HSPC with extremely high somatic mutation load.

To identify the processes underlying somatic mutation accumulation in fetal HSPCs, we determined the relative contribution of previously defined mutational signatures to the observed mutation spectra (see Methods)^23,24^. The mutation spectra between D21 fetal HSPCs and D21 post-infant HSPCs were significantly different (chi-squared test, *P* = 5.0 × 10^–4^) (Fig. 5a; Supplementary Fig. S7 and S8 online), which in part can be explained by a higher relative contribution of single base substitution signature 1 (SBS1) in fetal compared to D21 post-infant HSPCs (*P* < 5.0 × 10^–3^, permutation test) (Fig. 5b; Supplementary Fig. S8 online). The underlying mechanism of SBS1 is thought to be the spontaneous deamination of methylated cytosines, which likely reflects a cell cycle-dependent mutational clock^5,25^. Moreover, the relative contribution of the recently defined HSPC-specific mutational signature^4,22,24^ was less present in D21 fetal HSPCs, whereas it is predominant in D21 post-infant HSPCs^4^ (*P* < 5.0 × 10^–3^, permutation test) (Fig. 5b; Supplementary Fig. S8 online). In contrast, we did not find any difference between the mutation spectra of D21 fetal ISCs and D21 post-infant ISCs (*P* = 0.460, chi-squared test) (Supplementary Fig. S9 online), which is in line with a previous study^19^. The mutation spectra and relative contribution of mutational signatures between D21 and T21 fetal cells did not differ for HSPCs and ISCs. This indicates that the same mutational processes can explain the somatic mutations in D21 and T21 fetal stem and progenitor cells (Fig. 5a,b; Supplementary Fig. S9 online). Of note, the T21 fetal HSPC with a significantly higher mutation load compared to other T21 fetal cells did show contribution of an additional signature SBS18 (Fig. 5c), which has previously been associated with oxidative stress-induced mutagenesis^26^. Interestingly, an increase in the generation of radical oxygen species has been reported in T21 neurons, suggesting that ROS is preserved in several cell types in T21^27^. Our findings indicate that the increased mutation load in T21 fetal stem and progenitor cells is mostly caused by processes that are active during normal fetal development, suggesting that there is more activity of these mutational processes in T21 fetal stem and progenitor cells. However, the increased variance in mutation load might be explained by the activity of additional processes, such as oxidative stress-induced mutagenesis.

### T21 HSPCs display similar mutation load and patterns as DS-associated myeloid preleukemia

**Figure 6:**
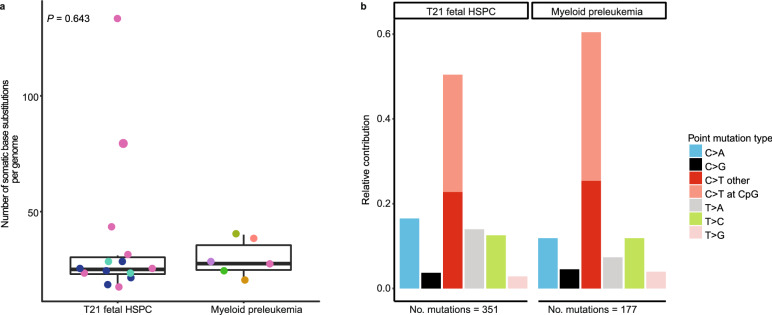
Somatic mutation patterns of preleukemic bulk blast cells from DS-associated myeloid preleukemia patients. (**a**) Comparison of the number of autosomal somatic base substitutions per genome per year for T21 fetal haematopoietic stem and progenitor cells (14 clones; 3 donors) and DS-associated myeloid preleukemia (6 donors). Points with the same color indicate single cells from the same subject. (linear mixed-effects model, two-tailed t-test). (**b**) 7-Spectrum of somatic base substitutions. The total number of somatic base substitutions is indicated.

We compared the somatic mutation load of preleukemic blast cells from 6 independent DS-associated myeloid preleukemia patients^11^ with those observed in the T21 fetal HSPCs and found similar numbers of mutations (*P* = 0.643, linear mixed-effects model) (Fig. 6a). As mutation accumulation in normal stem cells acts as a molecular clock^4,5^, our findings suggest that the DS-associated myeloid preleukemias arose during the period of fetal development that we assessed. In all preleukemic blast cells of DS-associated myeloid preleukemia patients we observed *GATA1* mutations (Supplementary table S3 online). However, no additional clonal cancer driver mutations were identified (Supplementary table S3). Moreover, we found no difference in the mutation spectra between T21 fetal HSPCs and DS-associated myeloid preleukemia, suggesting that SBS1, SBS5 and HSPC can explain the clonal somatic mutations in preleukemic blast cells of DS-associated myeloid preleukemia patients (*P* = 0.164, chi-squared test) (Fig. 6b, Supplementary Fig. S10 online). This observation indicates that no additional mutational processes are required to explain the somatic mutations in DS-associated myeloid preleukemia, besides those already active during normal fetal haematopoiesis.

## Discussion

In the present study we characterized mutation accumulation in individual HSPCs and ISCs of D21 and T21 fetuses. Several reports have demonstrated that T21 perturbs fetal haematopoiesis, which is explained by an imbalanced expression of genes involved in haematopoietic development^28–30^. Nonetheless, additional cancer driver mutations are needed for leukemic development^11,12^, suggesting that the HSPCs in the fetal liver of T21 fetuses are subjected to increased mutagenesis.

Our findings show that the somatic mutation rate in D21 fetal HSPCs is increased during normal fetal development compared to the post-infant mutation rate. Moreover, we found that the somatic mutation spectra vary between D21 fetal HSPCs and D21 post-infant HSPCs, indicating that HSPCs are exposed to different mutational processes during fetal development. Indeed, we found that the HSPC-specific signature is predominant in D21 post-infant HSPCs, while it is less present in D21 fetal HSPCs. This observation might reflect that HSPCs reside in a different niche during fetal development. In line with other studies, we suggest that HSPCs are more protected in the adult bone marrow niche^31^, because HSPCs are highly proliferative during fetal development in the liver and become quiescent after they have migrated to the bone marrow^32^. In line with this, D21 post-infant HSPCs have relatively less contribution of mutational signature SBS1, which likely reflects a cell cycle-dependent mutational clock^25^. This mutational process is predominantly active during fetal hematopoiesis and can explain the increased somatic mutation rate in D21 fetal HSPCs compared to D21 post-infant HSPCs. This suggests that the increased activity of SBS1 in D21 fetal HSPCs may contribute to the relatively higher incidence of leukemia in young children compared to young adults, since an increased mutation rate increases the chance to acquire a cancer driver mutation.

Moreover, fetal cells of T21 individuals, who are at risk of developing leukemia, show an even higher somatic mutation load, which was apparent before gastrulation. The increased mutation load in T21 fetal HSPCs was mostly caused by processes, which are active during normal fetal haematopoiesis. In addition, we showed that these mutational processes are sufficient to explain the somatic mutations in DS-associated myeloid preleukemia.

Previously, an association was found between aneuploidy and an increased mutation rate in several cancers^33^. Here, we have shown that an aneuploidy of chromosome 21 in human cells results in an increased somatic mutation load, indicating that the aneuploidy might be the first hit in these cancers. Moreover, it has been shown that aneuploid yeast strains show a mutator phenotype, which is suggested to be caused by deficient DNA repair in these strains^15^. Our study shows a similar phenotype in T21 stem and progenitor cells of T21 fetuses, independent of cell type. In line with this, several studies have reported that T21 cells of DS individuals have a decreased expression of various DNA repair genes^34,35^. This raises the hypothesis that the increased mutation load in T21 fetal stem and progenitor cells is caused by deficient DNA repair.

Many clinical features associated with DS are highly variable among individuals, such as cognitive impairment, the occurrence of heart defects as well as the development of leukemia^36^. At a molecular level, we also observed a higher variance in somatic mutation load between T21 fetal stem and progenitor cells. Intriguingly, enhanced cell-cycle and gene expression variability was previously shown in aneuploid yeast strains^37^, suggesting that also at a molecular level increased variance is a common characteristic among aneuploidies.

The increased somatic mutation rate in T21 fetal HSPCs will increase the chance to acquire an oncogenic driver mutation. However, the 500 times higher incidence of AMKL in DS children cannot solely be explained by the differences we observed in mutation accumulation between D21 and T21 fetal cells^8^. One T21 HSPC of a T21 fetus was an outlier and showed an extremely high somatic mutation load, which can partly be explained by SBS18. However, the somatic mutations observed in DS-associated myeloid preleukemia did not show contribution of SBS18. This observation indicates that the T21 HSPCs with extremely high somatic mutation load are not necessarily the cells which undergo clonal expansion and give rise to the DS-associated myeloid preleukemia. Therefore, other factors, such as cell–cell competition, selection and/or composition of the haematopoietic microenvironment are likely to also play a role in the development of leukemia in children with DS. These factors together with the observed increased somatic mutation load in T21 cells during fetal development may explain the increased risk of developing leukemia early in life for children with DS.

In addition, we used the somatic base substitutions in fetal HSPCs and ISCs to construct developmental lineage trees of four human fetuses. We found that the contribution of the developmental lineage branches to fetal tissue can vary between fetuses, which is independent of T21. Adult tissues predominantly show an asymmetric contribution of the developmental lineage branches to tissues, while we observed a symmetric as well as an asymmetric contribution of the developmental lineage branches to fetal tissues^4,21,22^. This difference may indicate that the contribution of developmental lineage branches to tissues can change during life, which might be explained by a lower death rate and/or a higher proliferation rate of cells of a developmental lineage branch later during development. Alternatively, this change might be the consequence of a bottleneck, which is also taking place during the early blastocyst-stage in the human embryo^38^.

Overall, our study provides insights in the mutation accumulation and developmental lineages during early embryogenesis and fetal haematopoiesis in normal and T21 human fetal development. These findings may contribute to the increased risk of leukemia early during life and the higher incidence of leukemia in Down syndrome.

## Methods

### Collection of human fetal material

The gestational age (GA) in weeks was determined by the measurement of first-trimester crown-rump length by ultrasonography. In this study we included 9 fetuses from GA week 12–17. The age in weeks after conception was determined by subtracting 2 weeks from the GA. Human D21 fetal material without medical indication from elective abortion material (vacuum aspiration) was collected in 0.9% NaCl (Fresenius Kabi) and stored on ice. Human T21 fetal material was obtained from pregnant women who decided to terminate pregnancy after a positive non-invasive prenatal testing (NIPT) result for trisomy 21, which was confirmed by cytogenetic confirmatory tests after invasive prenatal screening (chorionic villus sampling or an amniocentesis).

The fetal intestine, liver and long leg bones were isolated and stored in Advanced DMEM/F-12, supplemented with 1% penicillin/streptomycin, 1% GlutaMAX, and 1% HEPES 10 mM at 4 °C overnight and processed next day. A piece of fetal skin was frozen down at − 20 °C.

### Fetal liver and small intestine disassociation

Liver and intestine were disassociated into single cell solutions with collagenase digestion as follows: biopsies were minced and incubated with EBSS supplemented with 1 mg/ml collagenase type 1A (Sigma-Aldrich) and 0.1 mg/ml DNaseI (Sigma-Aldrich) for 30 min at 37 °C, while shaking. The tissue was further digested with a pipette if needed and incubated 10 minutes more. Subsequently, cells were filtered through a 70 um Nylon cell strainer. Single cell solutions were frozen down or further processed for culturing. Clonal ISCs WGS data from fetus F100916W15 and F100916W17 were obtained from Kuijk *et al*. 2019^19^.

### Clonal intestinal organoid cultures

Single intestinal cells were plated in Matrigel (Corning) droplets in limited dilution. Cells were cultured in human ISC organoid (CHIO) medium containing: 70% Advanced DMEM/F-12 supplemented with 1% penicillin/streptomycin, 1% GlutaMAX, and 1% HEPES 10 mM, 0.5 nM WNT surrogate (produced in house), 20% RSPOI conditioned medium (produced in house), 1 × B27 supplement (Thermo Fisher Scientific), 1 × Primocin (Invivogen), 1 : 1000 hES cell cloning & recovery supplement (Stemgent), 10 μM SB 202,190 (Sigma-Aldrich), 10 mM Nicotinamide, 1.25 mM N-acetylcysteine, 0.5 μM A83-01 (Tocris Bioscience), 10 μM Rho kinase inhibitor (Abmole), 10% noggin conditioned medium (produced in house) and 50 ng/ml hEGF (PeproTech). ISC cultures from fetus E080416, F100916W16 and F100916W17 were cultured in CHIO medium with 50% WNT conditioned medium and 100 ng/ml noggin (preprotech) as described before^19^. After 2–3 days small organoids appeared and medium was changed to human CHIO medium without hES cell cloning & recovery supplement. Clonal ISC cultures were derived by picking single organoids. Clonal organoid cultures were cultured in human CHIO medium without hES cell cloning & recovery and Rho kinase inhibitor. The cultures were expanded for 6–7 weeks until there was sufficient material for whole-genome sequencing.

### Isolation and culture of haematopoietic stem and progenitor cells

Mononuclear cells from fetal bone marrow were flushed out with Advanced DMEM/F-12, supplemented with 1% penicillin/streptomycin, 1% GlutaMAX, and HEPES 10 mM. Single liver cells or mononuclear cells from bone marrow were stained with an antibody cocktail to sort HSPCs as described before^18^. Single HSPCs (CD34 + ,lineage-, index sort) were sorted with the sony SH800S into round-bottom 384-well plates (Supplementary Fig. S1 online). HSPCs were cultured in StemSpan SFEM medium supplemented with growth factors as described before for 3–4 weeks before collection of the cells^18^.

### DNA isolation

DNA from skin biopsies, clonal ISC organoids and primary intestinal biopsies was extracted using Genomic tip 20/G (Qiagen). DNA from clonal HSPCs cultures, bulk preleukemic blast cells and T-cells was extracted using Qiamp DNA Micro Kit (Qiagen).

### DS-associated myeloid preleukemia samples

Viable frozen peripheral blood samples from 2 DS patients with DS-associated myeloid preleukemia were obtained from the biobank commission of the Princess Máxima Center for Pediatric Oncology. Mononuclear cells were stained with a cocktail of the following antibodies: CD3-BV650 (Biolegend, Clone UCHT1, 300467, 1:100), CD4-PerCP/Cy5.5 (Biolegend, Clone OKT4, 317427, 1:200), CD8-BV785 (Biolegend, Clone SK1, 344739, 1:100), CD19-BV421 (Biolegend Clone HCD14, 30224, 1:100) , CD14-AF700 (Biolegend, Clone HCD14, 325614, 1:100), CD56-BV711 (Biolegend, Clone HCD56, 318335, 1:50), CD34-APC (Biolegend, Clone 561, 343607, 1:50), CD38-PE (Biolegend, Clone HIT2, 303505, 1:50) , CD33-PE/Cy7 (Biolegend, Clone WM53, 303433, 1:100), CD117-PE-dazzle594 (Biolegend, Clone 104D2, 1:100), CD16-FITC (Biolegend, Clone 3G8, 302005, 1:100), CD20-FITC (Biolegend, Clone IVB201, 302303, 1:100). Bulk T-cells (CD3+/CD4+ and CD3+/CD8+) and preleukemic blast cells were sorted with the Astrios-EQ. Preleukemic blast cells were sorted according to the diagnostics flow data. Cell pellets were used for DNA isolation. Public data was used for the other 4 myeloid preleukemia samples^11^.

### Collection of post-infant data

Vcfs from cord blood and D21 post-infant HSPCs were obtained from Osorio *et al*. 2018^4^. Vcfs from D21 post-infant ISCs were obtained from Blokzijl *et al*. 2016^5^.

### Whole genome sequencing and read alignment

DNA libraries for Illumina sequencing were generated using standard protocols (Illumina) from 20–50 ng of genomic DNA isolated from clonally expanded haematopoietic blood and progenitor cells, preleukemic blast cells and T-cells. DNA libraries for Illumina sequencing from skin biopsies and clonal ISC organoids were generated from 500 ng DNA. All samples were sequenced (2 × 150 bp) using Illumina HiSeq X Ten sequencers or Nova sequencers to 30 × base coverage.

Version 2.6.0 of the Illumina Analysis Pipeline (https://github.com/UMCUGenetics/IAP) was used to align the reads and call variants similar to^5^. Copy numbers and b-allele frequencies were in concordance with the trisomy and sex state of all the bulks and clones. Initiation files are available upon request.

The bulk skin biopsies of N01, NR1 and NR2 were sequenced on both the Illumina HiSeq X Ten sequencers and the Nova sequencers. The resulting BAM files were merged using samtools merge^39^. The library (LB) and sample (SM) fields of the header were unified for each readgroup in the new bamfile.

### Base substitution filtering

Unique base substitutions, not present in bulk tissue were filtered similarly as described before^5^. We considered variants that were passed by VariantFiltration and had a GATK phred-scaled quality score (QUAL) ≥ 50 and MQ ≥ 60. Variants with multiple alternative alleles were removed. We excluded variant positions that overlapped with single-nucleotide polymorphisms (SNPs) in the SNP database (dbSNP) v137.b37, unless that variant had a COSMIC id (v76)^40,41^. In addition, we removed all variants that overlapped with an inhouse blacklist (available upon request). We only retained autosomal and X-chromosome variants. We additionally filtered on genotype quality (GQ), Depth (DP) and VAF. For bulk tissues we filtered on GQ ≥ 10 and VAF = 0, while for clones we used GQ ≥ 99 and VAF > 0.1. In both the bulk tissue and clone we used DP ≥ 20. Bulk skin was used to for all fetuses to control for germline variants, except for fetus MH3 and MH2, for these fetuses we used bulk intestine.

We used Dirichlet modeling to check the clonality of the clones. Subsequently, we removed all variants with a VAF below 0.3 to retain only the clonal substitutions. For T21 samples, we used a VAF ≥ 0.2 on chromosome 21, to account for the different expected VAF of clonal mutations. For X chromosomal variants in male donors we used VAF ≥ 0.99 and GQ ≥ 10 for clones and DP ≥ 10 for both clones and bulk tissues.

To identify variants that were (sub)clonally present in the bulk tissue, we first applied our filters as described above, but did not yet filter on QUAL, GQ, DP or VAF to generate a “somatic” vcf file. All obtained variants were characterized in the clones. For each variant we divided the clones into ‘present’ and ‘absent’ based on their genotype. We filtered the ‘present’ clones using the GQ, DP and VAF filters, we previously used for the clones, while we used the bulk GQ, DP and VAF filters for the ‘absent’ clones. If at least one ‘present’ clone and one ‘absent’ clone passed the filtering, the variant was retained. This way variants are retained that are both confidently present and confidently absent in at least one clone. Finally, all variants were manually inspected using IGV (v2.4.15)^42^.

### Indel calling/filtering

Indels were filtered similarly to SNVs, except for the following differences. Variants lying within 100b of a called germline indel were removed. We filtered on QUAL ≥ 250. For both bulk tissue and clones we filtered on GQ ≥ 99.

### Structural variant calling/filtering

We ran Gridss (v2.2.2) with bwa (v0.7.17) to detect structural variants (SVs)^43,44^. The output was filtered using a public pool of normal (3792v1) file from the Hartwig medical foundation (HMF) with the structuralvariantannotation (commit: d6173c3d9dd1fa314c91092b51920925b22268c6) R package and code modified from the HMF pipeline. In addition, we filtered for somatic SVs by only retaining variants in which at least one clone had a quality of 0. Next, we calculated VAFs and kept only breakpoints for which at least one clone had VAF ≥ 0.3. Then, all breakpoints were removed for which the partner was not kept. Finally, all variants were inspected by eye in IGV (v2.4.15)^42^. In the end, no SVs were observed.

### Driver mutations

The mutation load per clone could potentially be influenced by somatic driver mutations. We checked for the presence of driver mutations in the identified somatic mutations. A mutation was considered as possible driver if it met two requirements. First it needed to be annotated with “MODERATE” or “HIGH” effect by snpeff(v4.1) and second it needed to either have a COSMIC id (v76) or be located in a gene that was annotated as somatic in the Cosmic cancer gene census (v88)^41,45^.

The mutation load per clone could also be influenced by germline drivers. To identify potential predisposition variants, we started with the “somatic” vcf described before. We filtered the bulk tissue on GQ ≥ 50, DP ≥ 10 and VAF ≥ 0.3. Next, we removed all variants that had an allele count of more than 10 in either The ExAC (annotated via dbNSFPv2.9) or the GoNL (v5) database^46–48^. Furthermore, we only retained variants that were annotated with “MODERATE” or “HIGH” effect by snpeff(v4.1). Additionally, all variants were removed that did not overlap with a cancer-genes list from Zhang *et al*. 2015^48^. After manual inspection, none of the remaining variants were determined to be driver mutations.

### Mutation load accumulation

For each clone the total number of somatic mutations was extrapolated to the entire called genome, based on the surveyed fraction of the genome similar to^5^. For the comparison against post-infant data, we used only autosomal variants that were not present in corresponding bulk tissue in order to equally compare the mutations load with the same method. We calculated the lifelong mutation rate by dividing the mutation load with the age of the donor since conception. Next, a linear mixed-effects regression model was fitted to compare the mutation rates between the fetal, cord-blood and post-infant clones. A random intercept was modeled for the ‘donor’ to resolve the non-independence that results from having multiple measurements per donor. Significance values and 95% CI intervals were calculated using a two-tailed t-test.

To compare the mutation load between D21 and T21 fetal cells, we fitted a linear mixed-effects model where the extrapolated mutational load was fitted against the age of the donors, the trisomy state and the cell type. The trisomy state and the cell type were crossed. We allowed a different variance for D21 and T21 fetal cells. A random slope was modeled for the ‘donor’. We did not observe a difference in mutation load between the cell types or an interaction between cell type and trisomy state (Supplementary Fig. S3 online).

To test that the significance of our model was not dependent on its complexity, we fitted simplified versions of the model to our data. These also showed significant differences in mutation load between D21 and T21 (Supplementary Table S4 online). However, our original model had the best performance based on log likelihood, the Bayesian information criterion (BIC) and the Akaike information criterion (AIC).

To test whether the variance between D21 and T21 was different, a log-likelihood ratio test was used to compare our main model to a version with a single variance for both D21 and T21.

To test that the significance of our model was not dependent on a few single points, we performed a leave-n-out analysis. We iteratively removed each combination of n points from our data and fitted our model on the remaining data. We determined the distribution of p-values we got for each of our fixed variables. The difference between D21 and T21 fetal cells always remained significant for both n = 1 and n = 2 (Supplementary Fig. S4 online).

Outliers in the models were detected by calculating the odds of the standardized absolute residuals occurring under a standard normal distribution. Fdr values were calculated to correct for multiple testing.

To compare the number of early mutations between D21 and T21 fetal cells, we used mutations that were (sub-)clonally present in the bulk. As these mutations occurred before gastrulation, they should not be affected by donor age. Therefore, we fitted the same model as described previously on the mutation load of early mutations, but without age as an explanatory variable.

The Wilcoxon rank sum test with continuity correction was used to compared the number of dbs between D21 and T21 fetal cells. Mutations present in multiple clones of a single fetus were only counted in a single cell.

### Construction of developmental lineage tree

We created a binary mutation matrix with size CxM, where M is the number of mutations and C the number of clones. One and zero indicate presence and absence of a mutation in a clone. A row with only zeros was added to the matrix to root the tree. The pairwise distances between the clones where then calculated and a neighbourhood tree was generated.

Next, we calculated the VAFs in the bulk samples for all identified somatic mutations. For each developmental lineage branch, we took the mutations with a non-zero VAF and created a matrix containing the reference and alternative allele counts. Next, we performed a chi-square test on this matrix to see if the mutations had significantly different VAFs. This was the case for one branch, with three mutations, in which the mutations likely occurred in different cell divisions. After, we calculated the median VAF of the non-zero VAF mutations in each branch. We multiplied the median VAFs with factor 2, to get the contribution of these mutations to the bulk tissue.

To determine if the first two developmental lineage branches had different contribution to the bulk tissue, we calculated whether their VAFs, were significantly different. We summed up the reference and alternative allele counts of the non-zero VAF mutations in a branch. For each pairwise combination of branches we then performed a Fisher’s exact test.

### Mutational profile and signature analysis

For the comparisons against post-infant data, we used only autosomal variants, because X-chromosome variants were not called in the post-infant data. Furthermore, for the comparisons of D21 with T21 and the comparison of T21 with DS-associated myeloid preleukemia, we only used unique substitutions not present in the bulk tissue, because those mutations are most likely to have originated in the cell type of interest. Additionally, mutations subclonally present in bulk T-cells were not called in the DS-associated myeloid preleukemia samples. In other comparisons, all mutations were used. Because of the low mutational load per sample, mutations were pooled per category. Mutations occurring in multiple clones, were only counted once. Each mutational signature analysis, was performed by comparing two categories at a time. The T21 fetal HSPC with a high mutational load was not included in the T21 category, but was instead analyzed separately because it was an outlier. Chi-square tests were used to compare base substitution profiles. The mutational profiles were fitted to a matrix containing the COSMIC signatures and the recently discovered HSPC signature using Mutational Patterns^23^. To reduce overfitting, we applied an iterative reverse selection process. During each iteration the mutational profiles are fitted against the signatures. Next, the cosine similarity between the original and the reconstructed profile was calculated. The signature with the lowest contribution across the samples was removed. This process was repeated until the difference in cosine similarities between two iterations became more than the cutoff of 0.05.

To provide us with a confidence level of signature contributions, we performed a bootstrapped version of our signature refitting method, with 1,000 iterations. For the bootstrapping we resampled the mutational profiles with replacement.

By correlating the bootstrapped contribution of signatures, we were able to visualize how the selection of one signature influences the selection of another signature. SBS5 and SBS40, which have a cosine similarity of 0.83, are negatively correlated (Supplementary Fig. S8 online). This shows that a small difference in a mutational profile, can cause the signature refitting process, to select a different signature. Bootstrapping is thus necessary, to determine how confident the signature exposures are.

We used a permutation test to compare the mutational signatures between samples. This was done by permuting the mutation matrix 2000 times, while keeping the margins fixed. Refitting, was then performed on the permuted matrixes, using the signatures that were selected at least 50% of the time in the bootstrapping. This results in a distribution of exposures for each used signature. Next, we calculated per category and per signature, how often the permuted exposures were more extreme then the exposures calculated using the original matrix. This value was then divided by the number of permutations and multiplied by two, to generate a two-tailed p-value.

The previously described method to compare the signatures between two groups is rather stringent and removes signatures with a small contribution. As a result, SBS1 and SBS5 could no longer be detected in post-infant HSPCs, even though they were present in fetal HSPCs. Since the mutations caused by SBS1 and SBS5 can’t disappear, we decided to use a less stringent refitting method. Since SBS1, SBS5 and the HSPC signature were found to be present in HSPCs, we refitted the D21 HSPCs, T21 HSPCs, DS-associated myeloid preleukemia blasts and the post-infant HSPCs with only these signatures using the standard method from MutationalPatterns.

Mutational spectra, using previously defined mutational contexts, for the dbs and indel mutations were generated using inhouse R scripts.

We used modified versions of functions from the MutationalPatterns package to test whether there was an enrichment of mutations in regulatory regions, exons or genes.

### Ethical statement

The Medical Ethical Committee of the Leiden University Medical Center approved this study (P08.087). The study was performed in accordance with the guidelines and regulations of the Helsinki declaration and its later amendments or comparable ethical standards. Signed informed consent was obtained from participating women. DS-associated myeloid preleukemia samples were obtained after approval of the Biobank commission of the Princess Máxima Center for Pediatric Oncology. Signed informed consent was obtained from all parents.

## Supplementary information

Supplementary Figures.

Supplementary Tables.

## Data Availability

Data are available on EGA under accession number EGAS00001003982 and EGAS00001002886. Additionally, Vcfs and mutation matrixes are provided via the Github repository described in ‘Code availability’.

## Code availability

Code can be found on github at https://github.com/ToolsVanBox/Mutation_accumulation_T21.
