# Supplementary material for: Mutation accumulation and developmental lineages in normal and Down syndrome human fetal haematopoiesis
Source: Sci Rep. 2020 Jul 31;10:12991. doi: 10.1038/s41598-020-69822-1 (PMC7395765; doi:10.1038/s41598-020-69822-1)
Supplement: Supplementary file 1 — Supplementary Figures. [file 41598_2020_69822_MOESM1_ESM.docx]

Mutation accumulation and developmental lineages in normal and Down syndrome human fetal haematopoiesis

**Authors**

Karlijn A. L. Hasaart^1,†^, Freek Manders^1,†^, Marie-Louise van der Hoorn^2^, Mark Verheul^1^, Tomasz Poplonski^3^, Ewart Kuijk^4^, Susana M. Chuva de Sousa Lopes^5^ and Ruben van Boxtel^1,^*

^1^Princess Máxima Center for Pediatric Oncology and Oncode Institute, Heidelberglaan 25, 3584CS Utrecht, The Netherlands

^2^Leiden University Medical Center, 2333 ZC, Leiden, The Netherlands

^3^Princess Máxima Center for Pediatric Oncology, Heidelberglaan 25, 3584CS Utrecht, The Netherlands

^4^Center for Molecular Medicine, University Medical Center Utrecht and Oncode Institute, Universiteitsweg 100, 3584 CG Utrecht, The Netherlands

^5^Department of Anatomy and Embryology, Leiden University Medical Center, 2333 ZC, Leiden, The Netherlands

^†^These authors contributed equally

*Corresponding author: [R.vanBoxtel@prinsesmaximacentrum.nl](mailto:R.vanBoxtel@prinsesmaximacentrum.nl)

Fig. S1 Haematopoietic stem and progenitor cells isolation strategy.

Representative FACS strategy to sort haematopoietic stem and progenitor cells from fetal liver and bone marrow. Example data is from a trisomy 21 (T21) fetal liver


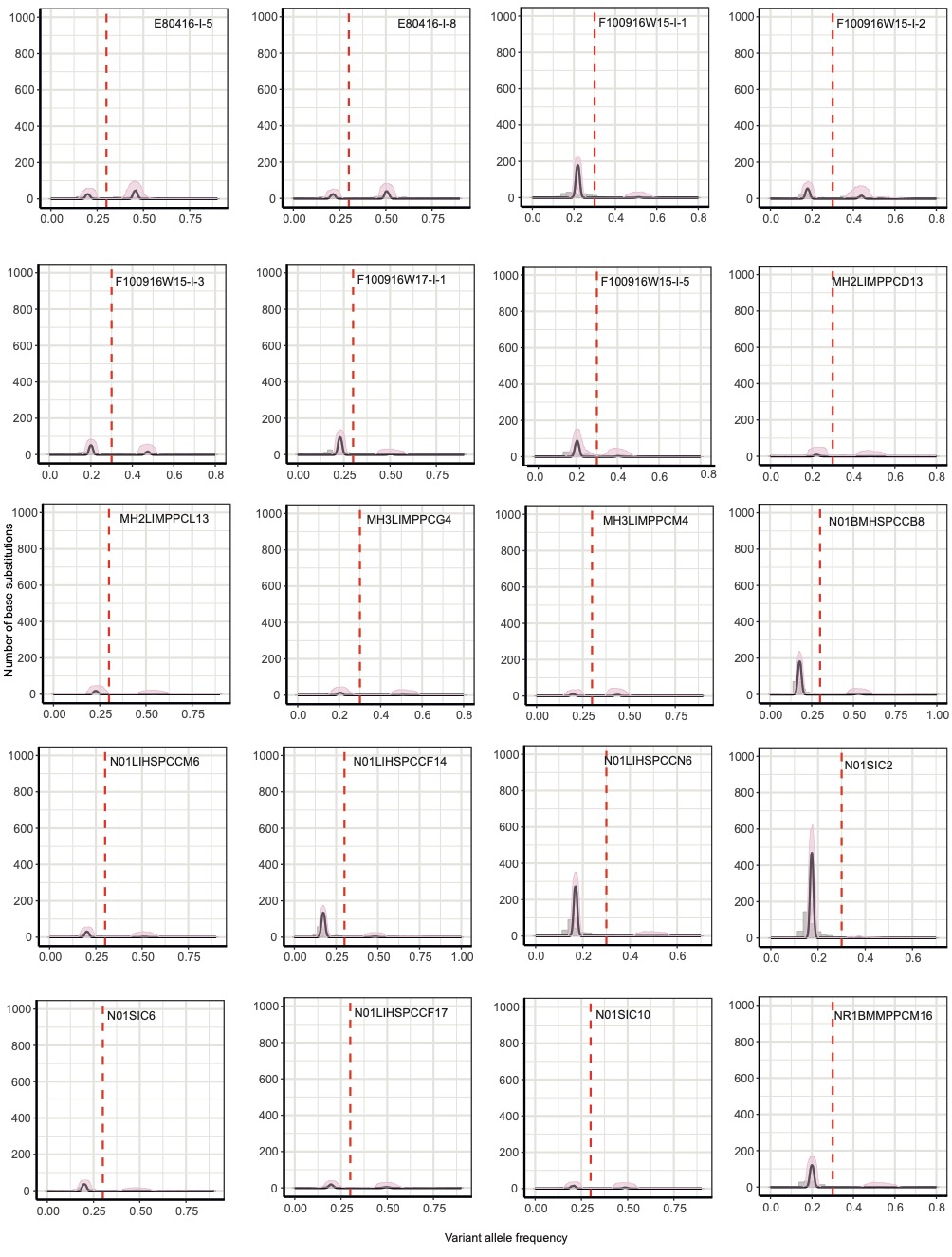


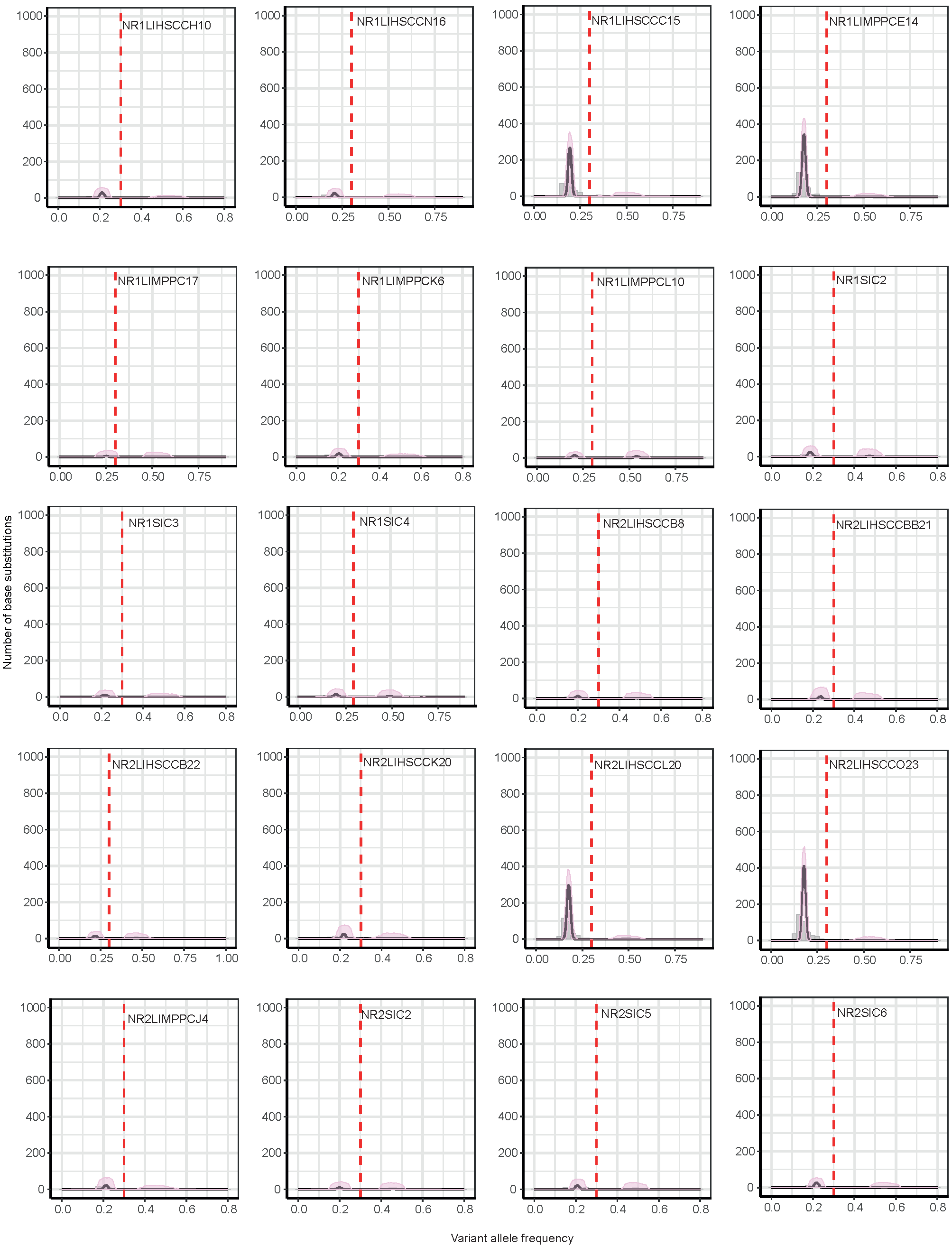


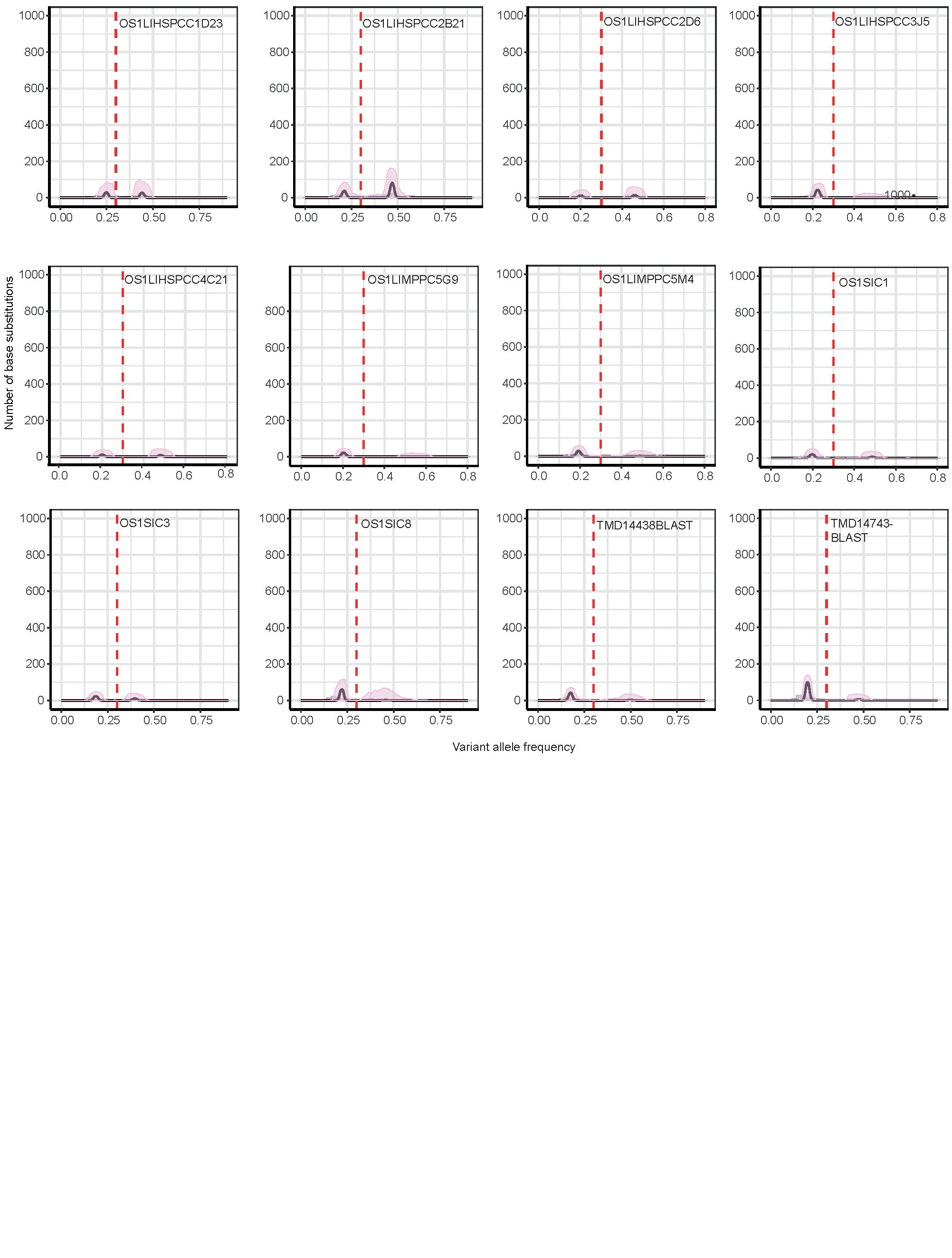


Fig. S2. Variant allele fractions (VAF) of base substitutions in sequenced clones.

Dirichlet modeling was used to determine the clonality of the cells. Histograms of the variant allele frequency of each sequenced sample to detect clonal single base substitutions. Clonal heterozygous mutations peak at VAF = 0.5. A threshold of VAF 0.3 was used to obtain mutations that were clonal and present in the original haematopoietic stem and progenitor cells or intestinal stem cells. Clonal mutations in the DS-associated myeloid preleukemia samples indicate the mutations present in the cell which underwent clonal expansion. Mutations acquired during or after clonal culture have lower VAFs and are therefore excluded. Shaded area represents the 95% posterior confidence intervals for the fitted distribution (pink area). In most samples, two clusters of mutations can be identified.

Fig. S3. Model parameters to determine somatic mutation load in fetal stem cells.

Model parameters of the linear mixed-effects model comparing the mutation load of disomy 21 (D21) vs trisomy 21 (T21). The model estimates of the explanatory variables are shown. Error bars represent 95% confidence intervals.

Fig. S4. Leave-n-out analysis on disomy 21 (D21) and trisomy 21 (T21) fetal stem and progenitor cells.

Each combination of n-points is iteratively removed and the linear mixed-effects model is calculated on the remaining data. The resulting P-values are shown for the different explanatory variables of the model. **a** n=1. **b** n=2.

Fig. S5. Indel and double base substitution (dbs) spectra.

**a** Contribution of the indicated indel mutation types to the indel mutation spectrum in disomy 21 (D21) and trisomy 21 (T21) fetal stem and progenitor cells. The top of the plot shows the indel size. The bottom shows how often the mutated bases are repeated in the genome. Mutations are pooled per category (D21 fetal: 28 clones; 5 donors, T21 fetal: 23 clones; 4 donors). **b** Contribution of the indicated dbs mutation types to the dbs mutation spectrum in D21 and T21 fetal stem and progenitor cells. The top of the plot shows the reference bases. The bottom of the plot shows their substitution. Mutations are pooled per category (D21 fetal: 28 clones; 5 donors, T21 fetal: 23 clones; 4 donors).

Fig. S6. Somatic indel mutation numbers in fetal stem and progenitor cells.

The number of somatic indels per genome plotted against the donor age (D21 fetal: 28 clones; 5 donors, T21 fetal: 23 clones; 4 donors). Dashed line: intestinal stem cells (ISC), full line: haematopoietic stem and progenitor cells (HSPC). P-value shows the difference between T21 and D21. (linear mixed-effects model, two-tailed t-test).

Fig. S7. 7-channel mutation spectra of each sequenced fetal clone.

Spectrum of point substitutions for each clone. The total number of point substitutions is

indicated.

Fig. S8. Differences in mutational patterns between disomy 21 (D21) fetal haematopoietic stem and progenitor cells (HSPCs) and D21 post-infant HSPCs.

**a** For each signature the percentage of bootstrap iterations (1000 iterations) in which this signature was present is shown. **b** Violin plot of the bootstrapped (1000 iterations) number of base substitutions that each mutational signature contributed to the mutational profiles. Thicker parts of the violin are supported by more iterations of the bootstrap. The widths are scaled to the maximum density of each signature. **c** The cosine similarity between the mutational profiles and the mean reconstructed profiles, based on the signature refitting are shown. **d** Heatmaps depicting the correlation of bootstrapped signature contributions are shown. **e** Signature permutation test (2000 permutations) for fetal vs post-infant D21 HSPCs. The bars show the mean relative signature contribution of the permutations. The error bars show the 2.5% and 97.5% quantiles. The dots show the actual measured relative signature contribution. (D21 Post-infant HSPC: n = 10924; 18 clones; 5 donors, D21 fetal HSPC: n = 353; 17 clones; 3 donors).

Fig. S9. Mutational patterns of disomy 21 (D21) post-infant, D21 fetal and trisomy 21 (T21) fetal intestinal stem cells (ISC).

**a** Spectra of point substitutions. The substitutions are pooled per category. (D21 Post-infant ISC: n = 21471; 14 clones; 9 donors, D21 fetal ISC: n = 340; 11 clones; 4 donors, T21 fetal ISC: n = 319; 9 clones; 3 donors). **b** The relative contribution of different mutational signatures to the spectra of point substitutions.

Fig. S10. Mutational signatures of preleukemic bulk blast cells from DS-associated myeloid preleukemia patients.

The relative contribution of different mutational signatures to the spectra of point substitutions. The substitutions from multiple samples were pooled together. (DS-associated myeloid preleukemia: n = 177; 6 donors).
